# Cuproptosis as a Potential Therapeutic Target for Steatotic Liver Disease

**DOI:** 10.3390/biom15111490

**Published:** 2025-10-23

**Authors:** Yujie Pan, Cheng Luo, Qitao Guo, Qifei Duan, Ziyan Wu, Yan Li

**Affiliations:** 1 School of Basic Medical Sciences, Yichun University, Yichun 336000, China; panyujie01340@163.com (Y.P.); luocheng@jxycu.edu.cn (C.L.); guoqitao017@163.com (Q.G.); dqifei19862532973@163.com (Q.D.); wzy1304542332@163.com (Z.W.); 2 School of Clinical Medicine, Yichun University, Yichun 336000, China; 3 School of Pharmaceutical Sciences, Yichun University, Yichun 336000, China

**Keywords:** copper, cuproptosis, steatotic liver disease, mitochondria, ferredoxin 1

## Abstract

Steatotic liver disease (SLD) has become one of the most prevalent chronic liver diseases, representing a significant health burden worldwide. The complex pathogenesis of SLD results in a lack of specific therapeutic targets and effective drug treatment modalities. Copper (Cu) is a trace element that plays a critical role in various physiological processes, particularly hepatic metabolism. Meanwhile, Cu overload can induce cellular toxicity, which is generally explained by its capacity to induce oxidative damage. In 2022, a novel form of programmed cell death, designated as cuproptosis, was identified. In essence, excess Cu ions bind to the lipoylated components of the tricarboxylic acid cycle, resulting in proteotoxic stress and subsequent cell death. The role of cuproptosis in the pathologies of Cu overload-induced diseases has gained considerable attention. However, the association between SLD and Cu overload, particularly cuproptosis, remains to be elucidated. This review provides a concise overview of cuproptosis. The significance of Cu overload in SLD, as well as the potential association between cuproptosis and SLD, is explored. This review aims to offer insights into the potential of cuproptosis as a therapeutic target for SLD.

## 1. Introduction

Hepatic steatosis refers to the accumulation of excessive lipids (≥5%) within liver parenchymal cells. Steatotic liver disease (SLD) is an umbrella term for a group of diseases characterized by hepatic steatosis. SLD can be caused by various etiologies, which encompass metabolic dysfunction-associated SLD, alcohol-related liver disease, metabolic dysfunction- and alcohol-related liver disease, and cryptogenic SLD [1,2]. There are three distinct stages of SLD: benign steatosis, steatohepatitis, and hepatic fibrosis or even cirrhosis [3]. The fundamental risk factors associated with the development of SLD encompass obesity, insulin resistance, and alcohol consumption. Patients with SLD frequently manifest additional features of metabolic syndrome, including type 2 diabetes [1]. Due to disparities in lifestyle, dietary habits, and alcohol consumption, the prevalence of SLD varies significantly across regions. A meta-analysis of global SLD incidence rates revealed a global combined prevalence of SLD to be 37.5%, with 33.6% of cases classified as metabolic dysfunction-associated SLD, 4.1% as metabolic dysfunction- and alcohol-related liver disease, and 2.2% as alcohol-related liver disease [4]. In addition to comprehensive public health approaches [5], the availability of a wider range of treatment options will help alleviate the global health burden caused by SLD [6]. Therefore, it is imperative to expose the underlying mechanisms of SLD development and progression in depth [7,8].

Copper (Cu) is the third most prevalent trace element in biological systems, following zinc and iron. It is predominantly present in the form of Cu^+^ (95%, intracellular) and Cu^2+^ (5%, extracellular) [9]. Free Cu^+^ and Cu^2+^ can undergo a reversible transformation into each other under specific conditions. In essence, the oxidizing environment (i.e., the presence of hydrogen peroxide (H_2_O_2_)) facilitates the oxidation of Cu^+^ to Cu^2+^, while the reducing environment (i.e., the presence of ascorbic acid, or reduced glutathione (GSH)) reduces Cu^2+^ to Cu^+^. The alterations in the valence state of Cu could induce the generation of reactive oxygen species (ROS), which has been demonstrated to induce oxidative damage [10]. The reactions that Cu catalyzes hydroxyl radical formation in the presence of H_2_O_2_ are referred to as Fenton-like reactions, while interactions between Fe^2+^ and H_2_O_2_ are designated as Fenton reactions [11].

In the human body, Cu typically binds to proteins such as metallothionein, ceruloplasmin, albumin, and cuproenzymes. This binding helps maintain the stability of Cu and prevents damage resulting from the oxidation state transition [12]. In addition, Cu functions as a cocatalytic factor, facilitating a range of biochemical reactions through transferring electrons as well as binding to corresponding proteins. It has been established that these reactions encompass redox homeostasis, mitochondrial function, lipid metabolism, and signaling molecules. Cu is indispensable for maintaining liver metabolism and other bodily functions [13,14,15,16].

Cu dyshomeostasis, deficiency, or overload disrupts important biochemical reactions, with the potential to result in metabolic disorders that may lead to disease [17,18,19]. In 2022, Tsvetkov et al. revealed a novel Cu-induced cell death mechanism designated as cuproptosis, which differs from the previously reported mechanisms such as oxidative stress and cell apoptosis. Specifically, excessive Cu leads to aberrant oligomerization of Cu-dependent thiocyanate proteins within the tricarboxylic acid (TCA) cycle. This results in protein toxicity stress, thereby triggering cell death [20]. The potential effects of cuproptosis on cardiovascular disease [21], kidney disease [22], and others [23,24] have been reviewed, while the specific role of cuproptosis in SLD remains to be elucidated. This review aims to elucidate the effects of Cu overload in the pathogenesis of SLD. The potential of cuproptosis as a therapeutic target for SLD will be proposed and discussed.

## 2. Literature Retrieval Strategy

The present review was primarily based on a thorough literature search in PubMed, Google Scholar, and Web of Science databases. The following keywords were retrieved: ‘steatotic’, ‘fatty liver’, ‘steatotic liver disease’, ‘fatty liver disease’, ‘steatohepatitis’; ‘copper’, ‘Cu’, ‘cuproptosis’, ‘copper-dependent cell death’, and ‘cuproptosis-related gene’. The initial screening was conducted using the title and abstract, with subsequent secondary screening performed through a full-text evaluation. At the time of the screening, no restrictions were imposed with regard to the year of publication. Nevertheless, preference was given to literature published more recently.

## 3. Cuproptosis

It is imperative to maintain optimal Cu concentrations within cells, a process referred to as homeostasis. Cu overload, defined as Cu concentrations exceeding the threshold required for maintaining homeostasis, will induce cellular stress and cytotoxicity. A prominent example is Wilson’s disease [25]. In 1978, Chan et al. reported that Cu could induce fibroblast death in culture [26]. Subsequent investigations have identified Cu-induced cell death in various cell types and have elucidated the involvement of autophagy [27], apoptosis [28], and ferroptosis [29] in these processes.

**Figure 1:**
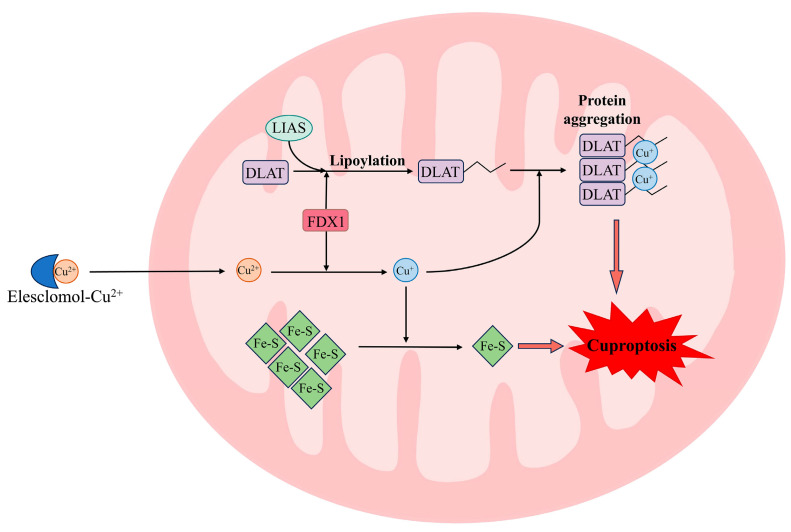
Cuproptosis pathway. Cu^2+^ is transported into mitochondria facilitated by Cu ionophore (e.g., elesclomol) and then is reduced to Cu^+^ by FDX1. Subsequently, Cu^+^ binds to the lipoylated TCA proteins, thereby inducing their oligomerization and proteotoxic stress. Additionally, Cu^+^ induces a loss of Fe-S cluster proteins, thereby resulting in mitochondrial dysfunction. Finally, the proteotoxic stress and mitochondrial dysfunction result in cell death. FDX1: ferredoxin 1; DLAT: dihydrolipoamide S-acetyltransferase; LIAS: lipoacylation synthase.

The biochemical characteristics associated with cuproptosis are distinct from other well-established types of cell death (Figure 1) [20]. Cu ionophores (e.g., elesclomol) facilitate the transportation and release of Cu into mitochondria. Subsequently, Cu binds to the lipoylated TCA proteins, thereby inducing their oligomerization and proteotoxic stress. Additionally, Cu induces a loss in Fe-S cluster proteins, thereby resulting in a destabilization of the electron transport chain (ETC) and mitochondrial dysfunction [30]. Finally, the proteotoxic stress and mitochondrial dysfunction result in cell death.

Cuproptosis exhibits a strong dependency on mitochondrial respiration. The inhibition of mitochondrial pyruvate uptake or electron transport, as well as hypoxia, significantly inhibits cuproptosis induced by Cu accumulation. The rate of basal or ATP-coupled cellular respiration remains unaltered during cuproptosis. However, the cell’s spare respiration is significantly diminished. Therefore, Cu targets pyruvate oxidation and the TCA process, but not the ETC components. This conclusion is validated by an investigation involving a genome-wide CRISPR/Cas9 loss-of-function screen in 489 cancer cell lines. Seven genes associated with lipoic acid metabolism were identified to play pivotal roles in cuproptosis, including ferredoxin 1 (FDX1), lipoacylation synthase (LIAS), lipoyl transferase 1 (LIPT1), dihydrolipoamide dehydrogenase (DLD), dihydrolipoamide S-acetyltransferase (DLAT), pyruvate dehydrogenase E1 subunit alpha 1 (PDHA1), and pyruvate dehydrogenase E1 subunit beta (PDHB). FDX1 has been identified as an upstream regulator of protein lipoylation modification [31]. LIAS, LIPT1, and DLD play pivotal roles in the lipoylation modification of proteins. DLAT has been identified as a protein target of lipoylation. DLAT, PDHA1, and PDHB are components of the pyruvate dehydrogenase complex, which is responsible for catalyzing the oxidation process of pyruvate. In addition, FDX1 functions as a regulator of Cu homeostasis, reducing Cu^2+^ to the more toxic Cu^+^. This process leads to the oligomerization of lipoylated proteins and the loss of Fe-S cluster proteins. Consequently, FDX1 acts as a crucial hub in cuproptosis [20].

## 4. Cu Overload in SLD

**Figure 2:**
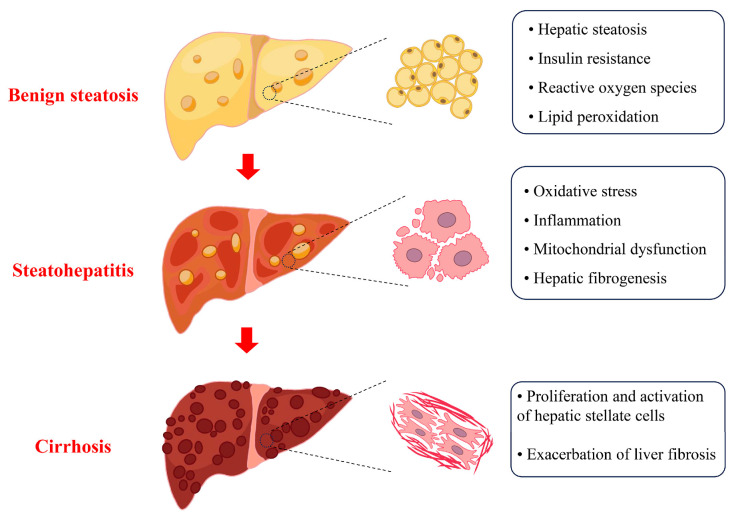
Progression of SLD. SLD ranges from early-stage benign steatosis to advanced steatohepatitis to eventual steatohepatitis-related cirrhosis. Each stage is accompanied by unique characteristics. For instance, hepatic steatosis and insulin resistance are present in the benign steatosis stage, while inflammation and fibrosis are present in the steatohepatitis stage. The cirrhosis stage is marked by the exacerbation of fibrosis.

Cu is essential in sustaining physiological functions. The liver, a vital organ responsible for Cu storage, plays a pivotal role in maintaining Cu homeostasis within the body. Meanwhile, it is also susceptible to Cu overload. An excess of Cu induced hepatic steatosis in zebrafish, modulated by liver X receptors α and sterol regulatory element binding protein 1 [32]. A cross-sectional study revealed a significant correlation between elevated serum Cu levels and the propensity to develop SLD [33]. This finding suggests that Cu overload may contribute to the development of SLD. However, it is imperative to note that SLD is a multifaceted disease, characterized by a spectrum of progressive stages, ranging from early-stage benign steatosis to advanced steatohepatitis, with the potential for subsequent steatohepatitis-related cirrhosis (Figure 2). The intricate relationship between Cu overload and SLD, particularly in regard to the differences in three distinct pathological stages, is not yet fully elucidated.

In the benign steatosis stage, which corresponds to the preliminary stages of SLD, the liver primarily exhibits substantial lipid accumulations (also known as hepatic steatosis), accompanied by insulin resistance. The process of lipotoxicity is initiated when the levels of abnormally accumulated lipids and their metabolites exceed the capacity of the liver. These alterations can result in a range of injuries, including endoplasmic reticulum stress, mitochondrial dysfunction, oxidative stress, and altered lysosomal permeability [34]. Hepatic steatosis has been observed under low-dose Cu exposure or in ATPase Cu transporters β (ATP7B) knockout mice and zebrafish models. The inherent inability of ATP7B knockout to effectively excrete Cu results in Cu accumulation within the liver and subsequent intrahepatic Cu overload [35,36]. The activation of the nuclear factor erythroid 2-related factor 2-mediated oxidative stress signaling axis and the recruitment of adipogenic gene promoters [19,37], as well as the pregnane X receptor/peroxisome proliferator-activated receptor γ signaling pathway, are identified as pivotal regulators of hepatic steatosis induced by Cu overload [38]. A significant positive correlation has been identified between dietary Cu intake and insulin resistance [39]. Cu accumulation promotes the generation of ROS, which impedes insulin signaling pathways, thereby contributing to the development of insulin resistance. Additionally, Cu toxicity promotes a cascade of events, including lipid peroxidation and the production of pro-inflammatory cytokines. These subsequent processes contribute to the exacerbation of insulin resistance [40]. The disruption of insulin function, characterized by insulin resistance, results in impaired ATP7B activity and subsequent intracellular Cu accumulation. This phenomenon in turn contributes to the exacerbation of insulin resistance. Additionally, another vicious cycle has been identified between the accumulation of lipids and the presence of insulin resistance. Both vicious cycles contribute to the progression of SLD [41,42].

The presence of lipid accumulation and insulin resistance in the benign steatosis stage promotes the progression to the steatohepatitis stage [43]. During this stage, the liver experiences a series of pathological changes, including lobular and/or portal inflammation, hepatocyte ballooning, and fibrosis [44]. The hallmark characteristics of steatohepatitis are mitochondrial dysfunction (e.g., oxidative stress), inflammatory response, and hepatocellular injury [45]. Hepatic oxidative stress and inflammation can be induced by Cu overload. The process is initiated by the lipid accumulation and insulin resistance [38,39], as well as the redox properties of Cu [46]. Mitochondria serve as the regulatory center of intracellular Cu homeostasis, controlling Cu storage [47] and efflux [48]. The mitigation of mitochondrial Cu accumulation, independent of the overall Cu loading of the liver, is efficacious in reversing Cu overload-induced mitochondrial dysfunction and improving the functional status of the liver [49]. Therefore, mitochondrial Cu accumulation appears to serve as a critical parameter in the progression of steatohepatitis.

Hepatic stellate cells are a type of interstitial cell in the liver and normally exist in a resting state. Under the stimulation of inflammation, injury, or other influence factors, hepatic stellate cells are activated and transform into collagen-producing myofibroblasts. This process can result in excessive collagen production, leading to fibrosis of the liver tissues and consequent liver dysfunction. Hepatic fibrosis facilitates the progression of steatohepatitis to the stage of cirrhosis. It is widely recognized as the most critical histologic predictor of mortality in patients with SLD [3]. Cu plays a major role in the development of organ fibrosis. Cu could stimulate the proliferation of hepatic stellate cells through oxidative stress, which in turn exacerbates the process of liver fibrosis [50].

## 5. Cuproptosis as a Potential Therapeutic Target for SLD

**Figure 3:**
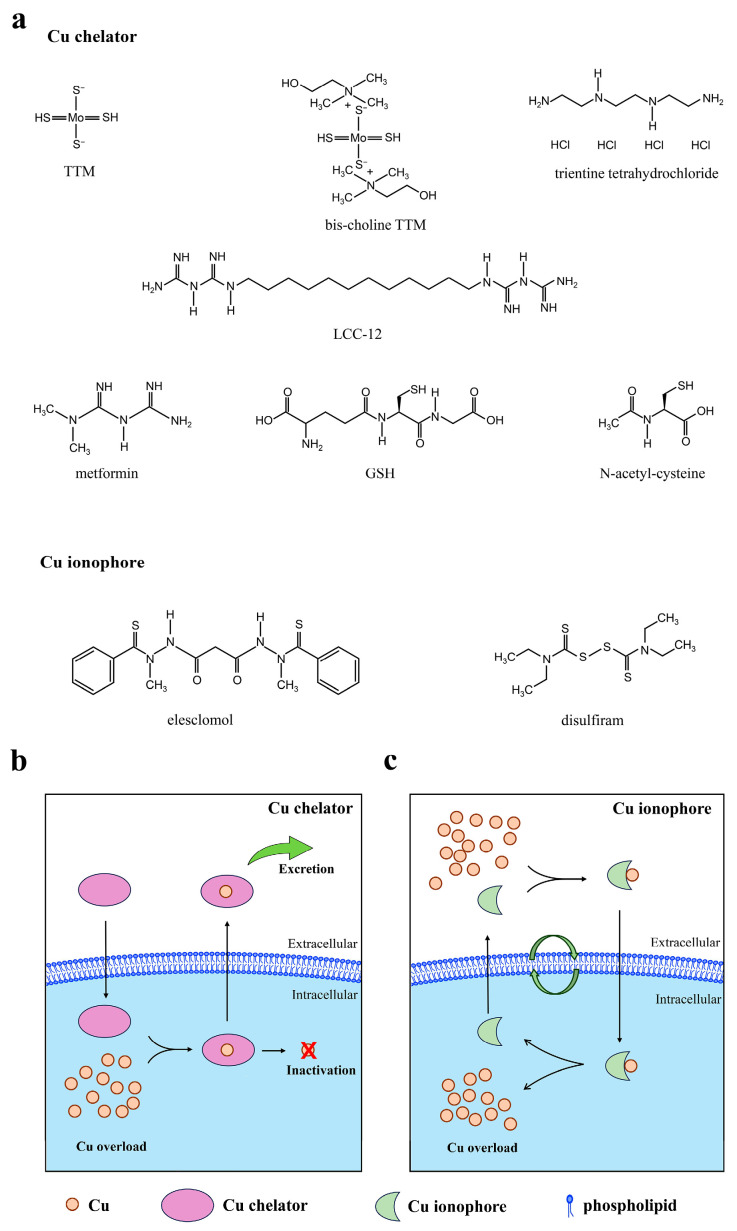
The chemical structures and mechanisms of Cu chelators and ionophores. (**a**) The chemical structures of common Cu chelators (TTM, bis-choline TTM, trientine tetrahydrochloride, LCC-12, metformin, GSH, NAC) and ionophores (elesclomol, disulfiram). (**b**) The mechanism of Cu chelators to mitigate Cu overload via Cu excretion and inactivation. (**c**) The mechanism of Cu ionophores to induce Cu overload and promote cuproptosis.

Evidence suggests a correlation between fluctuations in Cu levels and the progression of SLD [51,52,53]. The strategic regulation of Cu homeostasis emerges as a potential avenue for mitigating the progression of SLD (Figure 3) [54]. However, further investigation is required to ascertain the extent to which Cu concentrations are modulated in such cases and the specific mechanisms involved. Cu deficiency has been identified as a distinct risk factor for mortality in patients diagnosed with advanced liver disease [55]. However, the significance of Cu overload in the development of SLD cannot be overlooked, as previously explained. For instance, an investigation of patients with Wilson’s disease indicated that Cu accumulation may act as a crucial mediator of hepatic fat deposition. This finding demonstrated a clear correlation between hepatic Cu concentrations and the severity of hepatic steatosis, which is independent of metabolic factors [56]. This discrepancy can be attributed to the pivotal role of Cu homeostasis in maintaining liver health: either elevated or diminished Cu levels have detrimental effects [16].

Cuproptosis is induced by Cu accumulation. Despite the precise mechanism of cuproptosis remaining to be further elucidated, the present study hypothesizes that cuproptosis might be a pivotal factor in the progression of SLD. The expression of cuproptosis-related genes (DLD, DLAT, and PDHB) was found to be significantly higher in mice with SLD than in the normal controls [57,58,59]. Consequently, the regulation of intracellular Cu concentration to restore Cu homeostasis and prevent cuproptosis has the potential to emerge as a therapeutic approach for SLD. Cu chelators can bind with Cu ions directly, thereby reducing the concentration of free Cu ions in hepatocytes, which could prevent cuproptosis (Figure 3b). Moreover, Cu chelators can inhibit the activation of hepatic stellate cells by attenuating the accumulation of Cu within the intracellular environment, thus hindering the process of fibrosis. For healthy individuals at high risk of SLD or patients still in a reversible stage, mitigating the process of cuproptosis has the potential to prevent or delay the onset and progression of SLD.

Previous studies have shown that inducing apoptosis, necrosis, or aging of activated hepatic stellate cells can slow down the development of liver fibrosis [60,61,62]. Therefore, for patients at a critical stage of steatohepatitis-related cirrhosis, inducing cuproptosis of activated hepatic stellate cells might have the potential to alleviate the progression of cirrhosis. For example, Cu ionophores could facilitate the transport of Cu ions into hepatic stellate cells, thereby increasing the concentration of Cu ions and promoting cuproptosis (Figure 3c).

In addition to the primary mechanism of regulating Cu accumulation at the source, targeting cuproptosis-dependent metabolic pathways is also an effective strategy for regulating cuproptosis in SLD (Table 1).

### 5.1. Cu Chelators

The treatment of Wilson’s disease has historically focused on the modulation of Cu homeostasis, with a particular emphasis on Cu chelation therapy [63]. This therapeutic approach has been demonstrated to be efficacious in addressing the symptoms in the majority of patients with Wilson’s disease [64]. Cu chelators, including trientine tetrahydrochloride and ammonium tetrathiomolybdate (TTM), could be used to reduce Cu levels, although their final effects may vary [64,65]. Furthermore, bis-choline TTM (also named as WTX101 or ALXN1840), a Cu-protein-binding agent for oral administration, does not directly chelate Cu. Instead, it forms complexes with Cu and albumin and eliminates excess Cu from the liver and blood via bile, thereby treating Wilson’s disease [66,67]. Based on the previously delineated potential impact of Cu overload on the development of SLD, this treatment modality may offer significant insights to the management of SLD.

Glutathione (GSH) is a vital component of the human body and functions as both an antioxidant and a Cu chelator. *N*-Acetyl-cysteine (NAC) is a precursor for GSH biosynthesis, which replenishes GSH reserves. The depletion of intracellular GSH has been observed to result in atypically elevated sensitivity to cuproptosis, attributed to a deficiency in the capacity to eliminate toxic Cu ions [20]. The replenishment of intracellular GSH will impede cuproptosis, thereby hindering the progression of SLD. The efficacy of NAC in mitigating metabolic dysfunction-associated SLD-associated complications is well-established [68]. However, the precise mechanism remains to be elucidated [69]. It is reasonable that NAC functions as a prospective SLD mitigant through intracellular Cu chelation. This process can occur via a direct route or subsequent to conversion to GSH. This process leads to the inhibition of Cu-induced mitochondrial dysfunction, resulting in hepatoprotective effects. Undoubtedly, further mechanistic studies are warranted to resolve this enigma.

Targeting mitochondrial Cu inactivation may offer a promising approach to mitigate the progression of SLD. Metformin has emerged as a promising candidate molecule for the treatment of metabolic dysfunction-associated SLD [70,71]. Although clinical trials occasionally reported controversial results, which might be due to predominantly diminutive scale, cursory duration, and often discordant outcomes. More extensive randomized controlled trials of sufficient duration and incorporating histological endpoints remain paramount to fully ascertain the efficacy of metformin [70,71]. In addition, a more thorough investigation into the mechanisms through which metformin ameliorates SLD is imperative. Logie et al. revealed that metformin can form a pseudoaromatic planar ring structure with Cu. This reaction is facilitated by π-electron delocalization, leading to a reduction in available Cu within the mitochondria [72]. Stéphanie Solier et al. developed a metformin dimer, known as LCC-12, which was found to inactivate mitochondrial Cu. This process limits the production of key metabolites necessary to initiate and maintain the inflammatory state. The efficacy of LCC-12 was substantiated in diverse acute inflammatory murine models, ranging from LPS-induced endotoxemia and cecum ligation and perforation to SARS-CoV-2 virus infection models [73].

It is worth noting that the potential involvement of Cu deficiency in the pathogenesis of benign steatosis cannot be ruled out. Consequently, it is advised that patients monitor their blood Cu levels prior to the administration of therapy aimed at reducing Cu levels.

### 5.2. Cu Ionophores and Cu-Based Nanomedicine

Activation of hepatic stellate cells has been identified as the primary driver of liver fibrosis in SLD [74,75]. Inducing death of hepatic stellate cells represents a primary strategy for mitigating liver fibrosis and cirrhosis [75,76]. Therefore, the study of cuproptosis in the field of anti-tumor research has the potential to be utilized in anti-hepatic fibrosis, which may contribute to the management of SLD.

Cu ionophores are molecules that facilitate the delivery of Cu to cells, with the objective of modulating intracellular Cu ion levels. This approach has emerged as a promising strategy to address Cu deficiency or augment intracellular Cu levels in a controlled manner. The application of Cu ionophores to augment intracellular Cu levels and thereby initiate cuproptosis has garnered considerable research interest in tumor therapy [77]. Elesclomol and disulfiram are the most commonly used Cu ionophores in anticancer research. Nevertheless, the lack of specificity of these ionophores might facilitate the transportation of alternative metal ions and result in homeostatic imbalances. Moreover, the targeted delivery is another challenge. The employment of nanomedicine in tumor targeting and drug delivery proffers distinct advantages. The co-assembly of ionophore/Cu into nanomedicines will address the aforementioned problems more effectively. In addition, other types of Cu-based nanomaterials can also be applied to achieve intracellular Cu accumulation, thereby inducing cuproptosis [78].

### 5.3. Targeting Cuproptosis-Dependent Metabolic Pathways

Activated hepatic stellate cells demonstrate an augmented metabolic rate, which is characterized by the enhancement of mitochondrial respiration and elevated production of ATP. These alterations are consequences of the accelerated biological processes, in conjunction with the heightened energy requirements [79,80]. Cuproptosis is closely related to mitochondrial metabolism. Elevated mitochondrial metabolism has been observed to sensitize the cells to cuproptosis, while the inhibition of the mitochondrial electron transport chain or pyruvate uptake has the opposite effect [20]. Developing effective cuproptosis-based strategies for addressing hepatic fibrosis through metabolism regulation can draw lessons from the research conducted on cuproptosis in cancer treatment. For instance, by targeting the stability of FDX1, a critical regulator of cuproptosis, it was possible to enhance the sensitivity of gastric cancer to cuproptosis. The methyltransferase-like 16 (METTL16) catalyzes the addition of m^6^A modifications to FDX1 mRNA, thereby enhancing its stability and facilitating the accumulation of FDX1 protein. Sirtuin 2 (SIRT2) deacetylates METTL16, thereby inhibiting its methyltransferase activity. Consequently, the antitumor effects of cuproptosis are significantly enhanced by the inhibition of SIRT2 deacetylase activity by AGK2 in gastric cancers [81]. Ferroptosis, initially defined in 2012, is a form of iron metabolism disorder. The process is characterized by the presence of iron-dependent lipid peroxidation, which results in the accumulation of ROS. These reactive species are produced through the Fenton reaction [82]. Cross-talk between ferroptosis and cuproptosis has been observed, particularly in the context of metabolic pathway regulation [82,83]. GSH, for instance, can function as an antioxidant or a metal-chelating agent, exerting negative regulatory effects on ferroptosis and cuproptosis, respectively. In ferroptosis, GSH facilitates the maintenance of glutathione peroxidase (GPX4) activity, which is essential for the elimination of peroxidation free radicals. In cuproptosis, GSH acts as a Cu-chelating agent and is pivotal in constraining the activity of Cu^+^ ions and decreasing their binding to DLAT [82]. A recent study showed that combining ferroptosis inducers and cuproptosis initiators is effective in halting the progression of liver cancer [84]. Ferroptosis inducers such as sorafenib and erastin have been shown to inhibit the mitochondria-dependent degradation of the FDX1 protein. This, in turn, has been demonstrated to increase the levels of lipoylated proteins and to promote cuproptosis in primary hepatocellular carcinoma [84]. In addition, sorafenib also elicits ferroptosis in hepatic stellate cells via hypoxia inducible factor-1 α/solute carrier family 7 member 11 signaling, which in turn attenuates liver fibrosis [85]. The findings indicate that the targeted induction of these two novel cell death pathways has the potential to serve as a therapeutic approach for the treatment of liver fibrosis.

## 6. Conclusions and Future Perspectives

The identification of cuproptosis has provided new research directions for diseases associated with Cu dyshomeostasis. As a distinctive form of cell death, cuproptosis is initiated by elevated Cu concentrations within the mitochondria. Cu could bind to lipoylated TCA proteins, thereby inducing their oligomerization. Meanwhile, Cu could induce instability of Fe-S cluster proteins. These changes induced accumulation of protein toxic stresses, which ultimately lead to cell death. These processes involve pivotal components of the TCA cycle and are intricately linked to mitochondrial function.

In recent years, several studies have elucidated the molecular mechanisms of Cu homeostasis and its role in various diseases. While the precise mechanisms underlying Cu homeostasis in SLD remain to be fully elucidated, regulating Cu homeostasis has great potential as a novel therapeutic or interventional approach in addressing liver disease. This concept presents a novel perspective that facilitates the comprehension and management of the challenges encountered in the clinical management of SLD at the molecular level. However, the prevailing emphasis on Cu deficiency research in the SLD field has led to an unfortunate oversight concerning the significance of Cu overload in this disease. Consequently, it is imperative to explore the implications of Cu overload. This review presents the role of Cu overload in the progression of SLD, emphasizing the crucial function of Cu overload in the three stages of SLD: benign steatosis, steatohepatitis, and hepatic fibrosis or even cirrhosis. The modulatory mechanisms that underlie this process encompass the regulation of lipid metabolism disorders, insulin resistance, mitochondrial dysfunction, and fibrotic responses.

There exists an important correlation between elevated Cu levels and SLD. The review explores the potential implications of Cu overload and cuproptosis in the context of SLD pathogenesis, offering novel insights into the comprehension of the disease. Furthermore, observations indicate that cuproptosis-related genes manifest characteristic alterations in the progression of SLD. Consequently, it is recommended that cuproptosis be integrated into the conceptual framework for addressing the disease. For patients still in a reversible stage of SLD, reducing intracellular Cu concentration to restore Cu homeostasis and mitigate the process of cuproptosis has the potential to prevent or delay the onset and progression of SLD. On the other hand, an aberrant accumulation of Cu in hepatic stellate cells has been observed to promote the development of liver fibrosis. The induction of cuproptosis in hepatic stellate cells through further accumulation of Cu has the potential to reverse the pro-fibrotic effects of Cu, thereby converting them into anti-fibrotic effects. However, given the paucity of experimental validation, the correlation between cuproptosis and SLD remains indirect and speculative. This review has evolved from an examination of Cu overload to a discussion of cuproptosis, exploring the feasibility of a correlation between cuproptosis and SLD. It is imperative to acknowledge that the precise nature of the causal relationship between cuproptosis and SLD remains to be elucidated.

Research about cuproptosis is still in its nascent stages, and the precise mechanism remains to be fully elucidated. While the lipoic acid pathway has been identified as a pivotal mediator of cuproptosis, significant challenges persist in comprehending the intricacies of this novel form of cell death. It is imperative that researchers conduct in-depth studies to elucidate the phenotypes, reliable biomarkers, and the precise molecular mechanisms underlying cuproptosis in cases of Cu overload. Additionally, it is crucial to investigate the associations of cuproptosis with other forms of cell death. These efforts will contribute to a more profound understanding of the following aspects: firstly, the role of cuproptosis in the development of SLD; secondly, the development of personalized strategies to regulate Cu metabolism; and finally, the development of novel therapeutic interventions.

In summary, the review emphasizes the crucial role of Cu overload in SLD and establishes a connection with cuproptosis. Additionally, the review proposes prospective research directions, offering insights into therapeutic interventions targeting Cu and potential solutions to the challenges posed by SLD.

## Figures and Tables

**Table 1:** Cuproptosis as a potential therapeutic target for SLD.

Therapeutic Strategies	Mechanism	Targeting of SLD Stage
(1) Inhibition of Cu overload or cuproptosis	Excess Cu removal (Cu chelation) Targeting mitochondrial Cu inactivation	Benign steatosis Steatohepatitis
(2) Promotion of cuproptosis	Cu supplementation (Cu ionophore and Cu-based nanomedicine) Targeting cuproptosis-dependent metabolic pathways	Hepatic fibrosis/Cirrhosis

## Data Availability

No new data were created or analyzed in this study. Data sharing is not applicable to this article.

## Abbreviations

The following abbreviations are used in this review:

SLD	Steatotic liver disease
Cu	Copper
H_2_O_2_	Hydrogen peroxide
TCA	Tricarboxylic acid
ETC	Electron transport chain
FDX1	Ferredoxin 1
LIAS	Lipoacylation synthase
LIPT1	Lipoyl transferase 1
DLD	Dihydrolipoamide dehydrogenase
DLAT	Dihydrolipoamide S-acetyltransferase
PDHA1	Pyruvate dehydrogenase E1 subunit alpha 1
PDHB	Pyruvate dehydrogenase E1 subunit beta
ATP7B	Atpase Cu transporters β
ROS	Reactive oxygen species
TTM	Ammonium tetrathiomolybdate
GSH	Glutathione
NAC	*N*-acetyl-cysteine
METTL16	Methyltransferase-like 16
SIRT2	Sirtuin 2
GPX4	Glutathione peroxidase 4
